# Nontraumatic Spontaneous Bilateral Basal Ganglia Hemorrhage: A Rare Case Report

**DOI:** 10.7759/cureus.11299

**Published:** 2020-11-02

**Authors:** Tariq A Shaheed, Nicholas Glover, Safwan Alboiny

**Affiliations:** 1 Hospital Medicine / Internal Medicine, UC Davis Health, Lodi, USA; 2 Graduate Medical Education / Emergency Medicine, Desert Regional Medical Center, Palm Springs, USA; 3 Neurology, Kaiser Permanente, Roseville, USA

**Keywords:** intracerebral hemorrhage (ich), neurology, intracranial bleed, mri, head ct

## Abstract

A 62-year-old man with a past medical history of uncontrolled hypertension, tobacco abuse, and type 2 diabetes mellitus (DM) presented to the emergency department due to worsening confusion over the last 24 hours as reported by a friend. A CT brain without contrast was obtained, which demonstrated a bilateral intracerebral hemorrhage (ICH). Spontaneous bilateral intracerebral hemorrhage is an exceedingly rare condition with only 30-40 reported cases*.* This patient had a non-traumatic ICH, without focal neurological deficits on presentation. The patient had no complications while hospitalized despite the imaging findings. Clinicians should keep a broad differential similar to causes of spontaneous non-traumatic unilateral ICH, including uncontrolled hypertension, tumor mass, coagulopathies, and vasculopathies. Although brain CT is the most appropriate study in the acute setting, MRI is the gold standard for definitive diagnosis and should be performed urgently to further characterize the lesions. Clinicians should be aware of non-traumatic ICH complications, which include aspiration pneumonia, quadriparesis, hemiparesis, and recurrent stroke. Management is supportive mainly by reducing risk factors for complications, including blood pressure control, aspiration precautions, reversing coagulopathies, frequent neurological checks, and consultation with multiple disciplines such as neurosurgery or neurointerventional radiology.

## Introduction

A spontaneous unilateral intracerebral hemorrhage (ICH) is a well documented medical condition; however, a spontaneous bilateral ICH is exceedingly rare, with only 30-40 reported cases [1]. The rare incidence of this disease process makes this topic specifically interesting. We discuss a curious case of a man who suffered a nontraumatic spontaneous bilateral ganglia ICH captured on medical imagery and is followed in our outpatient practice.

## Case presentation

A 62-year-old man with a reported past medical history of uncontrolled hypertension was seen at our hospital emergency department due to worsening confusion over the last 24 hours with an acute exacerbation of confusion, witnessed by friend 30 minutes prior to the initial evaluation. Upon arrival, he had no specific complaints. He was on a daily 81 mg aspirin and multiple blood pressure medications. He denied any other past medical or surgical history. He had no family history of diabetes; he had no history of coagulopathy but was unsure about any family history of any neurological disease or cancer. Social history was significant for smoking two cigarettes a day for 15 years but quit 10 years ago. On exam two hours after initial emergency department evaluation, he was alert and oriented to person, place, time, and event, but did elicit some confusion and had a Glasgow Coma Scale (GCS) of 14 (eye-opening - 4, verbal response - 4, motor response - 6). Cranial nerves II-XII were grossly intact, yet horizontal gaze was noted to be mildly abnormal. The remainder of his neurological exam was unremarkable with pupils equal, round, and reactive to light; sensation to light touch intact to upper and lower extremities; 5/5 grip strength as well as in the upper and lower extremities; deep tendon reflexes (DTRs) 2+/4 in upper and lower extremities bilaterally; no dysdiadochokinesia appreciated; and no truncal ataxia. The heart was found to be at a regular rate but bradycardic. No heart murmur was noted, and blood pressure was not elevated.

**Figure 1:**
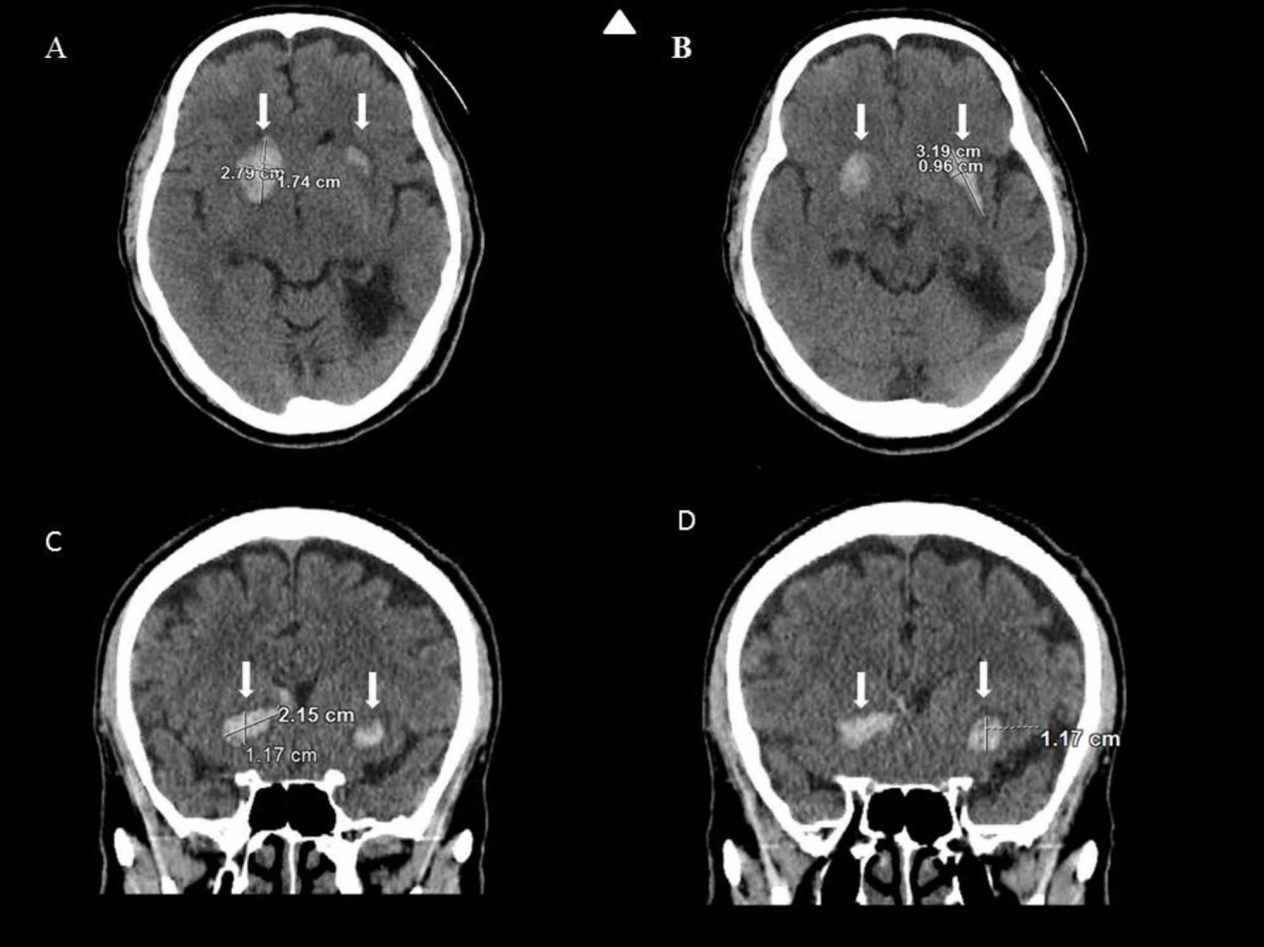
Brain CT images Brain CT shows parenchymal hemorrhage of the right basal ganglia, measuring up to 2.8 cm, with extension into the frontal horn of the right lateral ventricle. The same brain CT shows intraparenchymal hemorrhage of the left lentiform nucleus as well as chronic encephalomalacia of the left temporal lobe. Arrowhead indicates top. A small arrow indicates areas of interest, which are hyperattenuation on non-contrast CT study consistent with ICH. A: CT WO transverse; hyperattenuation measures 2.79 cm x 1.74 cm B: CT WO transverse; hyperattenuation measures 3.19 cm x 0.96 cm C: CT WO coronal; hyperattenuation measures 2.15 cm x 1.17 cm D: CT WO coronal; hyperattenuation measures 1.17cm ICH - intracerebral hemorrhage; WO - without contrast

STAT computed tomography (CT) brain without contrast showed parenchymal hemorrhage of the right basal ganglia and intraparenchymal hemorrhage of the left lentiform nucleus (Figure 1). Comprehensive metabolic panel (CMP), complete blood count (CBC), and lipid panel were all within normal limits, except HDL of 25 mg/dL. A urine drug screen was negative. EKG demonstrated bradycardia at 55 bpm, but otherwise a normal EKG. A portable chest X-ray showed no acute cardiopulmonary disease.

**Figure 2:**
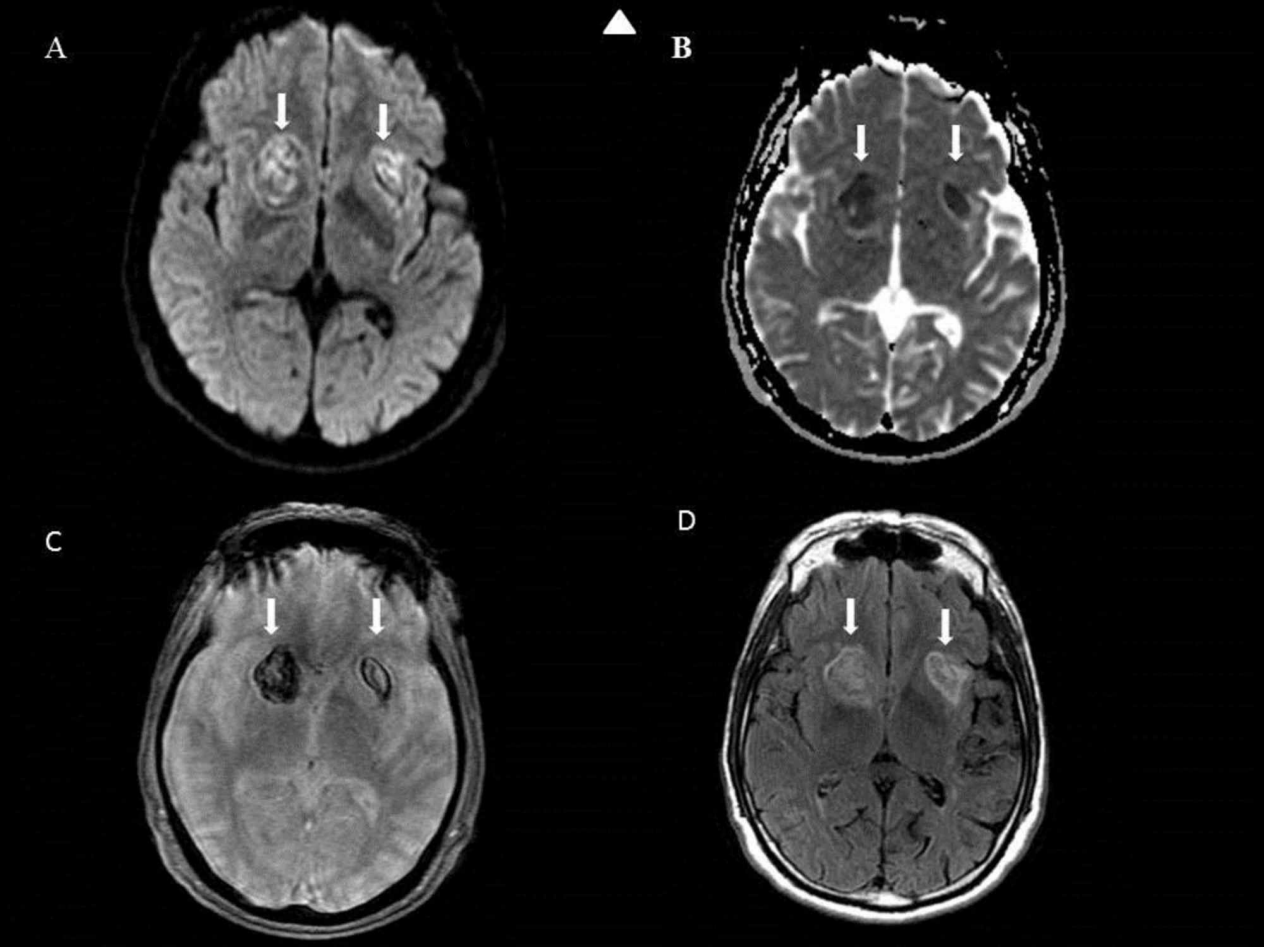
MRI Brain Brain MRI demonstrates 1) no definite evidence of acute ischemic infarct; 2) subacute hematoma in the bilateral basal ganglia greater on the right with extension into the right frontal bone as on the previous CT; 3) small T1 and flair hyperintensity with possible slight enhancement at the posterior aspect of the left Sylvian fissure, possibly a small vascular lesion; 4) evidence of mild to moderate chronic ischemic foci in the white matter. Arrowhead indicates top. Arrows indicate areas of interest on this MRI brain study with and without contrast. Correlates with clinical assessment of hypertensive subacute ICH in bilateral basal ganglia. A: MRI WWO contrast DWI B: MRI WWO contrast ADC C: MRI WWO contrast GRE D: MRI WWO contrast T2 flair ICH - intracerebral hemorrhage; WWO - with and without contrast; DWE - diffusion-weighted images; ADC - apparent diffusion coefficient; GRE - gradient recalled echo

The patient was admitted to the ICU overnight and recovered an additional 48 hours on the telemetry floor. His hospital course was unremarkable except for one dose of hydralazine PRN, and his systolic blood pressure remained less than 160. Neurosurgery was consulted, and no neurosurgical intervention was indicated. A magnetic resonance imaging (MRI) brain scan was obtained on the first day of admission demonstrated that hematoma was subacute in the bilateral basal ganglia, as seen on the initial scan (Figure 2) with no evidence of arteriovenous malformation (AVMs). MR neck angiogram with and without contrast showed evidence of mild atherosclerotic luminal narrowing of the right internal carotid origin and a hypoplastic left vertebral, which is usually developmental. An echocardiogram with a bubble study demonstrated a left ventricular ejection fraction greater than 60% with no wall abnormalities, no significant valvular regurgitation or vegetations, no thrombi identified, and no bubble contrast shunt from the interatrial septum. The patient was discharged after reaching the maximal benefit of hospital stay and is following in a hospital-associated outpatient clinic with the primary care provider (PCP) and neurology.

## Discussion

Incidence

Spontaneous unilateral intracerebral hemorrhage (ICH) is reported to be 24.6 per 100,000 person-year; however, a spontaneous bilateral ICH is exceedingly rare, with only 30-40 reported cases, and clinicians should be aware of the management, complications, and risk stratification recommendations. Of the previously reported cases of multiple spontaneous simultaneous ICH, 10.5% died, 10.5% was considered severely disabled, 2.6% was left in a vegetative state, 10.5 % walked with a cane, and 5.2% had a good recovery [2].

Differential diagnosis

This patient presented with subtle changes in behavior and mental state but had no focal deficits on the exam, highlighting the importance for clinicians to consider spontaneous ICH in patients with mental status changes. The main causes of spontaneous ICH include hypertension, tumor mass, coagulopathies, and vasculopathies [2]. Risk factors such as a prior stroke, hypertension, and advanced age can cause both unilateral as well as bilateral intracerebral hemorrhage. An expanded differential diagnosis for bilateral abnormalities of the basal ganglia and thalamus includes cerebral amyloid angiopathy,** **which places patients at risk for multiple spontaneous intracerebral hemorrhages [2]. Even more rare differential diagnosis categories include poisoning, metabolic diseases, vascular diseases, degenerative diseases, inflammatory and infectious diseases, neoplasms [3].

Management

Complications of a nontraumatic spontaneous bilateral basal ganglia ICH include aspiration pneumonia, quadriparesis, hemiparesis, and recurrent stroke [2]. Management is largely supportive by reducing risk factors for complications, including blood pressure control with a systolic blood pressure goal of 140 if there is no evidence of carotid stenosis and 160 if there is evidence of stenosis. Computed tomography angiography (CTA) neck or carotid doppler are the best studies to rule out carotid stenosis. Elevation of the head of the bed greater than 30 degrees assists in reducing the risk of aspiration. Early consultation with neurosurgery is paramount to evaluate any indications for any neurosurgical intervention [4].

## Conclusions

Spontaneous bilateral ICH is a rare event and can present severely with quadriplegia or, as in this case, with mild neurological deficits. Advanced age, history of hypertension, and prior stroke are the most common risk factors for spontaneous bilateral ICH, but subsequent follow up is necessary to rule out more rare disease processes. Although the brain CT is the most appropriate initial study in the acute setting, MRI is the gold standard for definitive diagnosis and should be performed urgently to further characterize the lesions. CT angiography is a critical test to assess for vascular malformation, which could require intervention.
